# Supplementary material for: The long noncoding RNA H19 regulates tumor plasticity in neuroendocrine prostate cancer
Source: Nat Commun. 2021 Dec 21;12:7349. doi: 10.1038/s41467-021-26901-9 (PMC8692330; doi:10.1038/s41467-021-26901-9)
Supplement: Supplementary file 3 — Description of Additional Supplementary Files [file 41467_2021_26901_MOESM3_ESM.pdf]

Description of Additional Supplementary Files

File Name: Supplementary Data 1
Description: SNPs Significantly Associated to Prostate Cancer Risk Near H19

File Name: Supplementary Data 2
Description: Differential Expression Analysis P-values

File Name: Supplementary Data 3
Description: Experimentally Verified Transcription Factor Binding Sites within H19

File Name: Supplementary Data 4
Description: TomTom Predicted Transcription Factor Binding Sites in H19

File Name: Supplementary Data 5
Description: JASPER Predicted Transcription Factor Binding Sites For AR/SOX2/SOX9 in H19

File Name: Supplementary Data 6
Description: JASPER Predicted Transcription Factor Binding Sites AR and SOX2 Highlighted in H19 DNA Sequence

File Name: Supplementary Data 7
Description: Peak occupancy- consensus anotation for H3K27me3 in V16D/CTL cells

File Name: Supplementary Data 8
Description: Peak occupancy- consensus anotation for H3K27me3 in V16D/H19 cells

File Name: Supplementary Data 9
Description: Peak occupancy- consensus anotation for H3K4me3 in V16D/CTL cells

File Name: Supplementary Data 10
Description: Peak occupancy- consensus anotation for H3K4me3 in V16D/H19 cells

File Name: Supplementary Data 11
Description: Differential binding analysis (n=3) results for H3K4me3

File Name: Supplementary Data 12
Description: Differential binding analysis (n=3) results for H3K27me3

File Name: Supplementary Data 13
Description: Bivalent genes for V16D/CTL cell line

File Name: Supplementary Data 14
Description: Bivalent genes for V16D/H19 cell line

File Name: Supplementary Data 15
Description: Differential methylation genome wide analysis of shH19 vs Control OWCM-155.

File Name: Supplementary Data 16
Description: Comparison of Differential methylation analysis of shH19 vs Control OWCM-155 with differential methylation of NEPC vs. AdPC from Beltran et al 2016.

File Name: Supplementary Data 17
Description: List of primers (5'-3') used for qPCR analysis

File Name: Supplementary Data 18
Description: Original Ct values of H19 in various samples.

File Name: Supplementary Data 19
Description: Chip-seq alligned reads generated from BAM files that are in peaks in both sample and input.

File Name: Supplementary Data 20
Description: Chip-seq average sequencing depth reads per sample.
