# Supplementary material for: The long noncoding RNA H19 regulates tumor plasticity in neuroendocrine prostate cancer
Source: Nat Commun. 2021 Dec 21;12:7349. doi: 10.1038/s41467-021-26901-9 (PMC8692330; doi:10.1038/s41467-021-26901-9)
Supplement: Supplementary file 24 — Reporting Summary [file 41467_2021_26901_MOESM24_ESM.pdf]

nature research

Corresponding author(s): Colin Collins, PhD and Andrew S. Kraft, MD

Last updated by author(s): Aug 31, 2021

Reporting Summary

Nature Research wishes to improve the reproducibility of the work that we publish. This form provides structure for consistency and transparency in reporting. For further information on Nature Research policies, see our Editorial Policies and the Editorial Policy Checklist.

Statistics

For all statistical analyses, confirm that the following items are present in the figure legend, table legend, main text, or Methods section.

n/a	Confirmed	
☐	☒	The exact sample size (n) for each experimental group/condition, given as a discrete number and unit of measurement
☐	☒	A statement on whether measurements were taken from distinct samples or whether the same sample was measured repeatedly
☐	☒	The statistical test(s) used AND whether they are one- or two-sided<br>*Only common tests should be described solely by name; describe more complex techniques in the Methods section.*
☐	☒	A description of all covariates tested
☐	☒	A description of any assumptions or corrections, such as tests of normality and adjustment for multiple comparisons
☐	☒	A full description of the statistical parameters including central tendency (e.g. means) or other basic estimates (e.g. regression coefficient) AND variation (e.g. standard deviation) or associated estimates of uncertainty (e.g. confidence intervals)
☐	☒	For null hypothesis testing, the test statistic (e.g. F, t, r) with confidence intervals, effect sizes, degrees of freedom and P value noted<br>*Give P values as exact values whenever suitable.*
☒	☐	For Bayesian analysis, information on the choice of priors and Markov chain Monte Carlo settings
☐	☒	For hierarchical and complex designs, identification of the appropriate level for tests and full reporting of outcomes
☐	☒	Estimates of effect sizes (e.g. Cohen's d, Pearson's r), indicating how they were calculated

Our web collection on statistics for biologists contains articles on many of the points above.

Software and code

Policy information about availability of computer code

Data collection	No software was used. All clinical patient sequencing and microarray data (Table 1) was available in-house through previous publication submissions. Please see methods "Clinical patient samples and cohorts" for publication references
Data analysis	All previously published algorithms or databases used are listed within our methods including any parameters selected. Data was analyzed using Microsoft Excel Office 2016, GraphPad Prism 9, ImageJ 1.52a, Incucyte organoid growth software, Integrated Genome Browser 9.0.1, JASPAR, Gene Set Enrichment Analysis, UCSC GrCH37/hg19 genome browser, Bismark, MethylKit (v. 1.11), R (v. 3.4), MACS2, ChIPQC Bioconductor package, DiffBind R Bioconductor package, ChIPseeker R Bioconductor package, clusterProfiler Bioconctor R package, R-Chie

For manuscripts utilizing custom algorithms or software that are central to the research but not yet described in published literature, software must be made available to editors and reviewers. We strongly encourage code deposition in a community repository (e.g. GitHub). See the Nature Research guidelines for submitting code & software for further information.

Data

Policy information about availability of data

All manuscripts must include a data availability statement. This statement should provide the following information, where applicable:

- Accession codes, unique identifiers, or web links for publicly available datasets
- A list of figures that have associated raw data
- A description of any restrictions on data availability

All clinical patient sequencing and microarray data (Table 1) was available in-house through previous publication submissions. Initial description, interrogation, and results for these datasets can be are available in their referenced publications. Please see methods "Clinical patient samples" for publication references. For access to these datasets, please use the following accession codes in reference to cohort labels from Table 1: VPC (PRJEB19256, PRJEB21092, PRJEB6530, PRJEB9660), BCCA (EGAD00001004139/EGAC00001000914), MCI (GSE46691), MCII (GSE62116), and JHMI (GSE104786). WCM1, WCM2, WCDT, and GRID cohorts require

original study author permission and are restricted access patient cohorts. Sequencing preformed in this study was deposed under accession codes GSE182914 (eRRB – Methylation Sequencing) and GSE122112 (ChIP Sequencing). The source data for Figs. 3I, 3K, 4H, 4I, 4K, 5D, 6B, 6C, 6D, 6F are provided as a Source Data file. All other data supporting the findings of this study are available within the article and its supplementary information files and from the corresponding author upon reasonable request. A reporting summary for this article is available as a Supplementary Information file.

Field-specific reporting

Please select the one below that is the best fit for your research. If you are not sure, read the appropriate sections before making your selection.

☒ Life sciences ☐ Behavioural & social sciences ☐ Ecological, evolutionary & environmental sciences

For a reference copy of the document with all sections, see nature.com/documents/nr-reporting-summary-flat.pdf

Life sciences study design

All studies must disclose on these points even when the disclosure is negative.

Sample size	For patient cohort analysis, sample size was not chosen based on statistics and solely on availability of patient specimens. For in vitro and in vivo experiments, an appropriate number of technical and biological replicates were generated under similar conditions yielding statistically significant results between groups. Results with no significant difference after being tested multiple times were deemed non significant.
Data exclusions	No data was excluded.
Replication	Experiments were performed multiple times (mostly in triplicates) with biological replicates unless otherwise noted in the methods or figure legends.
Randomization	For in vivo experiment, mice were randomized into groups before subcutaneous injection.
Blinding	Blinding was performed for analysis including cell proliferation and organoid growth analysis, imaging for immunohistochemistry staining, xenograft tumor measurement (weight and volume). For molecular analyses, data collection and analysis were performed by different investigators, using arbitrary sample codes whenever possible, to minimize bias. Investigators were not blinded to group allocation during remaining in vitro data collection and analysis. For in vivo experiment, to minimize bias during subcutaneous injection, the injection was performed by a trained UofA animal technician (working independently from study authors) who was provided the cells.

Reporting for specific materials, systems and methods

We require information from authors about some types of materials, experimental systems and methods used in many studies. Here, indicate whether each material, system or method listed is relevant to your study. If you are not sure if a list item applies to your research, read the appropriate section before selecting a response.

Materials & experimental systems

n/a	Involved in the study	
☐	☒	Antibodies
☐	☒	Eukaryotic cell lines
☒	☐	Palaeontology and archaeology
☐	☒	Animals and other organisms
☒	☐	Human research participants
☒	☐	Clinical data
☒	☐	Dual use research of concern

Methods

n/a	Involved in the study	
☐	☒	ChIP-seq
☒	☐	Flow cytometry
☒	☐	MRI-based neuroimaging

Antibodies

Antibodies used	SOX2 (cat no. sc-365823, Santa Cruz Biotechnology), H3K27me3 (cat no. 9733, Cell Signaling Technology), EZH2 (cat no. 5246, clone no. D2C9, Cell Signaling Technology), NSE (sc-271384, Santa Cruz Biotechnology), Synaptophysin (cat no. sc-365488, Santa Cruz Biotechnology), CHGA (cat no. 60893S, Cell Signaling Technology), BRN2 (cat no. 12137, clone no. D2C1L, Cell Signaling Technology), H3 (cat no. 4499, clone no. D1H2, Cell Signaling Technology), Androgen receptor (cat no. 5153, Cell Signaling Technology), H3K4me3 (cat no. ab8580, Abcam), P53 (2527, Cell Signaling Technology), Rb (9313, Cell Signaling Technology), PSA (cat no. sc-7316, Santa Cruz Biotechnology), HRP conjugated anti-β-actin (Cat. no. A3854, Sigma), SOX2 (cat no. 23064, Cell Signaling Technology), CK8 (cat no. ab53280, clone no. EP1628Y, Abcam), Ki-67 (cat no. 9027, clone no. D2H10, Cell Signaling Technology), H3K4me3 (Cat no. C15410003, Diagenode), HRP-linked mouse IgG (Cat. no. NA931V, GE Healthcare Life Sciences) and HRP-linked rabbit IgG (Cat. no. NAV934V, GE Healthcare Life Sciences).
Validation	Each primary antibody validated as per previous publications and reports available at the manufacturer's website. We also performed in-house validation for some of the antibodies. The validation statement for each primary antibody has been taken from the manufacturer's website or datasheet. Specific application of these antibodies in the manuscript has also been listed. Validation

statements are detailed as follows:

SOX2 (cat no. sc-365823, Santa Cruz Biotechnology): Validated by the manufacturer using Positive Controls: F9 cell lysate, H69AR whole cell lysate or C6 whole cell lysate. Application: Western blot

H3K27me3 (cat no. 9733, Cell Signaling Technology): Validated by the manufacturer using extracts from HCT113, NIH/3T3, COS, C6 cells (positive controls). Application: Western blot
For Chromatin immunoprecipitations (ChIP), the antibody was validated by the manufacturer (as shown in the datasheet) by performing ChIP with cross-linked chromatin from HeLa cells and either H3K27me3 antibody, or Normal Rabbit IgG, using SimpleChIP Enzymatic Chromatin IP Kit (Magnetic Beads). The enriched DNA was quantified by real-time PCR using GAPDH Exon 1 Primers, RPL30 Exon 3 Primers, MyoD1 Exon 1 Primers, and MYT-1 Exon 1 Primers. The amount of immunoprecipitated DNA in each sample is represented as signal relative to the total amount of input chromatin, which is equivalent to one.

EZH2 (cat no. 5246, clone no. D2C9, Cell Signaling Technology): Validated by the manufacturer using extracts from COS-7, Neuro2A and MCF7 cells. In-house validation was also performed using cells with EZH2 knockdown. Application: Western blot

NSE (sc-271384, Santa Cruz Biotechnology): Validated by the manufacturer using Positive Controls: IMR-32, T98G, H4, Neuro-2A, EOC 20 whole cell lysate. Application: Western blot

Synaptophysin (cat no. sc-365488, Santa Cruz Biotechnology): Validated by the manufacturer using Positive Controls: Rat brain tissue extract. In house validation was performed by using mouse pancrease as positive control. Application: Western blot

CHGA (cat no. 60893S, Cell Signaling Technology): Validated by the manufacturer using extracts from CHGA positive TT cells and CHGA negative HuH-6 cells. Application: Western blot

BRN2 (cat no. 12137, clone no. D2C1L, Cell Signaling Technology): Validated by the manufacturer using extracts from SK-N-MC, SKN-MEL-28, A375 cells (positive controls). Application: Western blot

H3 (cat no. 4499, clone no. D1H2, Cell Signaling Technology): Validated by the manufacturer using extracts from HeLa, NIH/3T3, COS, C6 cells (positive controls) and detects endogenous levels of total Histone H3 protein, including isoforms H3.1, H3.2, and H3.3. This antibody also detects the Histone H3 variant CENP-A. The antibody has not been shown to cross-react with other core histones. Application: Western blot

Androgen receptor (cat no. 5153, Cell Signaling Technology): Validated by the manufacturer using AR positive (LNCaP, MCF-7) and AR negative (PC3, DU145) cell lines. Application: Western blot
For validation in Chromatin immunoprecipitation (ChIP) experiments, ChIP were performed with cross-linked chromatin from LNCaP cells grown in phenol red free medium and 5% charcoal stripped FBS for 3 d then treated with dihydrotestosterone (DHT, 10 nM) for 4 hours and Androgen Receptor (D6F11) XP Rabbit mAb, using SimpleChIP Plus Enzymatic Chromatin IP Kit (Magnetic Beads). Results by the manufacturer demonstrated binding across KLK2 and enrichment at KLK3 promoters, known target genes of Androgen Receptor. Application: ChIP

H3K4me3 (cat no. ab8580, Abcam): Validated by the manufacturer using Calf thymus histone preparation (nuclear lysate). Application: Western blot

P53 (2527, Cell Signaling Technology): Validated by the manufacturer using extracts from 293 and COS cells. Further in-house validation was performed by using cells with p53 knockdown. Application: Western blot

Rb (9313, Cell Signaling Technology): Validated by the manufacturer using extracts from Jurkat cells and WI-38 cells. Further in-house validation was performed by using cells with Rb knockdown. Application: Western blot

PSA (cat no. sc-7316, Santa Cruz Biotechnology): Validated by manufacturer by Western blot analysis of PSA expression in βME-treated human prostate tissue extract. Application: Western blot

HRP conjugated anti-β-actin (Cat. no. A3854, Sigma): Validated by manufacturer by Western blot analysis of proteins isolated from various cell extracts. Application: Western blot

SOX2 (cat no. 23064, Cell Signaling Technology): Validated by manufacturer in Chromatin immunoprecipitation (ChIP) experiments, ChIP were performed with cross-linked chromatin from mES (mouse embryonic) cells and Sox2 (D9B8N) Rabbit mAb, using SimpleChIP Enzymatic Chromatin IP Kit (Magnetic Beads). Results by the manufacturer demonstrated binding across POU5F1/OCT4 and NANOG, known target genes of Sox2. Application: ChIP

CK8 (cat no. ab53280, clone no. EP1628Y, Abcam): Validated by manufacturer by staining Formalin/PFA-fixed paraffin-embedded sections) of human thyroid carcinoma tissue sections, human breast adenocarcinoma tissue sections and mouse liver tissue sections, after following the blocking and epitope retrieval steps. Application: Immunohistochemistry

Ki-67 (cat no. 9027, clone no. D2H10, Cell Signaling Technology): Specifically developed for immunohistochemical analysis of Ki-67. Validate by the manufacturer by staining paraffin embedded tissue sections for colon carcinoma, breast carcinoma and ovarian senous adenocarcinoma, after following the blocking and epitope retrieval steps. Application: Immunohistochemistry

H3K4me3 (Cat no. C15410003, Diagenode): Valiadted for ChIP by the manufacturer as follows (as shown in the datasheet)- ChIP was performed on sheared chromatin from 1 million HeLaS3 cells using 1 µg of the Diagenode antibody against H3K4me3 (cat. No. C15410003). The IP'd DNA was subsequently analysed on an Illumina Genome Analyzer. Library preparation, cluster generation and sequencing were performed according to the manufacturer's instructions. The 36 bp tags were aligned to the human genome using the ELAND algorithm. Result shows the peak distribution along the complete sequence and a 600 kb region of the X-chromosome and in two regions surrounding the GAPDH and EIF4A2 positive control genes, respectively. These results clearly showed the

	enrichment of the H3K4 trimethylation at the promoters of active genes. Application: ChIP

Eukaryotic cell lines

Policy information about cell lines

Cell line source(s)	HEK293T, LNCaP, C4-2B, VCAP, PC3, LASCPC-01 (PMID: 27050099), and NCI-H660 cell lines were purchased from the American Type Culture Collection (ATCC). The cell lines were cultured as recommended by the ATCC. Dr. Amina Zoubeidi provided V16D, 42D, and 42F cells, which were cultured as described previously (PMID: 27784708). LAPC-4 cells (RRID: CVCL_4744) were generously provided by Dr. Charles Sawyers (Sloan Kettering Memorial Center, NY, USA) and were cultured as described previously (PMID 9095173).
Authentication	Cell lines were authenticated at the UAGC (University of Arizona Genetics Core) by using autosomal STR (short tandem repeat) profiling.
Mycoplasma contamination	Regular testing of mycoplasma contamination was performed in these cell lines using MycoAlert™ Mycoplasma Detection Kit (LT07-118, Lonza) and only mycoplasma free cells were used for experimentation.
Commonly misidentified lines (See ICLAC register)	No misidentified cell lines were used in the study

Animals and other organisms

Policy information about studies involving animals; ARRIVE guidelines recommended for reporting animal research

Laboratory animals	The immunodeficient NOD scid gamma (NOD.Cg-Prkdcscid Il2rgtm1Wjl/SzJ) male mice were intially purchased from Jackson Laboratories, Bar Harbor, Maine and breeding colonies were maintained in the University of Arizona animal care facility from where the mice used in the study were procured. The mice were housed in ventilator racks (RAIR IVC system, Lab Products Seaford, DE) and maintained under specific pathogen-free conditions. The mice were fed NIH-31 irradiated pellets (Tekland Premier, Madison, Wisconsin) and sterile water was freely available. Daily light cycles were kept consistent in the animal facility (12h light and 12h dark). Cages were changed fully once a week. Sentinel mice were screened monthly by ELISA serology for mycoplasma, mouse hepatitis virus, pinworms, and Sendai virus and tested negative. 5-7 week old mice were subcutaneously injected for OWCM-155 organoid xenograft implantation.
Wild animals	Study did not involve use of wild animals.
Field-collected samples	Study did not involve use of field collected samples
Ethics oversight	All animal experiments were performed in accordance with protocols approved by The University of Arizona Institutional Animal Use and Care Committee.

Note that full information on the approval of the study protocol must also be provided in the manuscript.

ChIP-seq

Data deposition

☒ Confirm that both raw and final processed data have been deposited in a public database such as GEO.

☒ Confirm that you have deposited or provided access to graph files (e.g. BED files) for the called peaks.

Data access links *May remain private before publication.*	Access to excel files for the called peaks are provided in the Supplementary tables.
Files in database submission	FASTQ files for ChIP-sequencing of H3K27Me3 and H3K4Me3 from 3 V16D and 3 V16D/H19 cells samples. Bigwig, peak files, and data analysis files are also included.
Genome browser session (e.g. UCSC)	No longer applicable

Methodology

Replicates	3 replicates were used for each sample and histone mark interrogated with a respective input for each replicate.
Sequencing depth	Data described in methods. Average sequencing depth was approximately 14.3 million reads and sequencing depth per sample is listed in Supplementary Table 20
Antibodies	H3K4me3 (Diagenode, Catalogue # C15410003) and H3K27me3 (Cell Signaling Technologies, Catalogue # 9733).
Peak calling parameters	Peaks for each replicate (n=3) from each cell line, were called from BAM alignment files using MACS2 with default parameters. Narrow peaks were called for the H3K4me3 mark and broad peaks were called for the H3K27me3 mark (using the parameter --broad). The input BAM file for each replicate was used for both histone marks.

Data quality

The peaks assessed for coverage , fraction of reads in peaks (FRiP) scores and signal distribution using the ChIPQC Bioconductor package. ChIPSeq result quality satisfied and exceeded Encode standards. The samples were then interrogated for peak occupancy and differential binding using the DiffBind R Bioconductor package. For occupancy analysis, Consensus peaks were generated (using the replicates for each mark and cell line via dba.overlap) with an overlap rate of 0.66 and bivalent/biphasic regions were defined as regions of overlap between H3K4me3 and H3K27me3 marks for each cell line. Significant differentially bound regions for each histone mark, between V16D-H19 (n=3) vs V16D (n=3) cell lines were defined as regions having FDR < 0.05 using the default differential binding algorithm (dba.analyze) in the DiffBind R Bioconductor package. For Gene Ontology (GO) classification and enrichment, (pAdjustMethod = "BH", pvalueCutoff = 0.05, qvalueCutoff = 0.05) analysis for Biological Processes (BP), Molecular Functions (MF) and Cellular Component (CC) was performed using the clusterProfiler Bioconctor R package.

Software

Differentially bound regions, consensus peaks and bivalent/biphasic regions were annotated for proximity to genes using the ChIPseeker R Bioconductor package. All downstream analysis and plotting for ChIPSeq data analysis was generated using the R statistical platform.
