# Supplementary material for: The long noncoding RNA H19 regulates tumor plasticity in neuroendocrine prostate cancer
Source: Nat Commun. 2021 Dec 21;12:7349. doi: 10.1038/s41467-021-26901-9 (PMC8692330; doi:10.1038/s41467-021-26901-9)
Supplement: Supplementary file 1 — Supplementary Information [file 41467_2021_26901_MOESM1_ESM.pdf]

Supplementary Figure 1

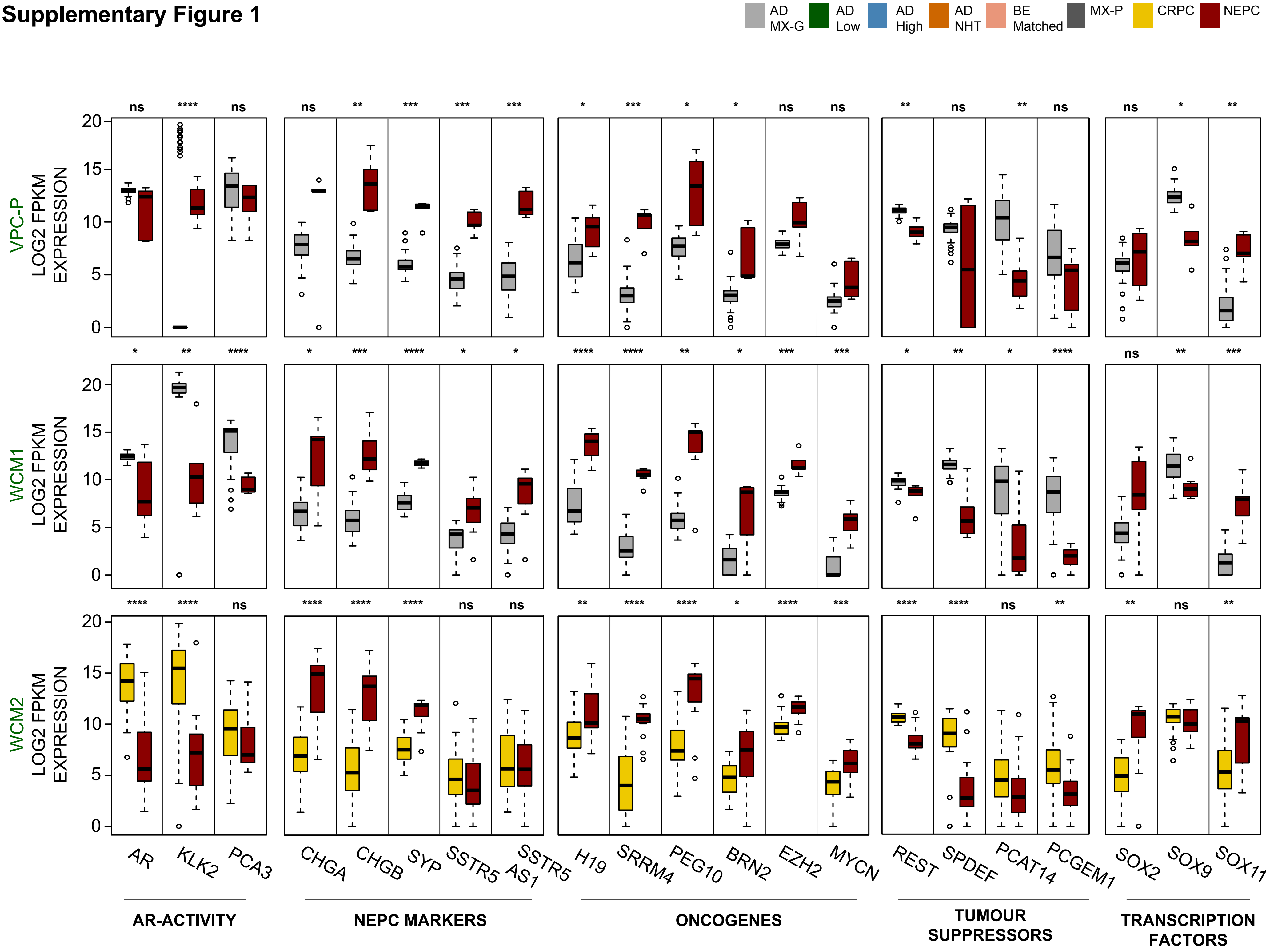

Supplementary Figure 2

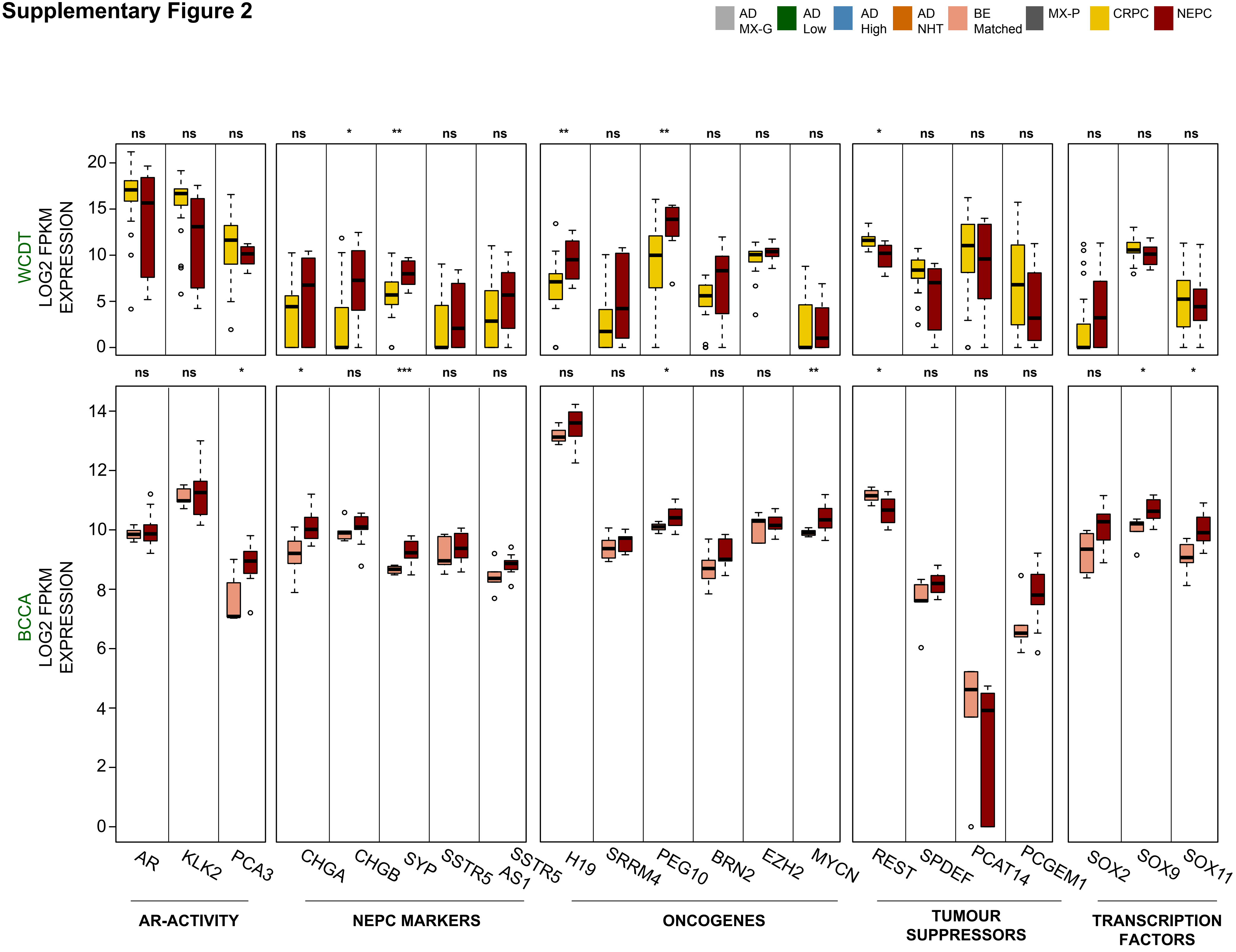

Supplementary Figure 3

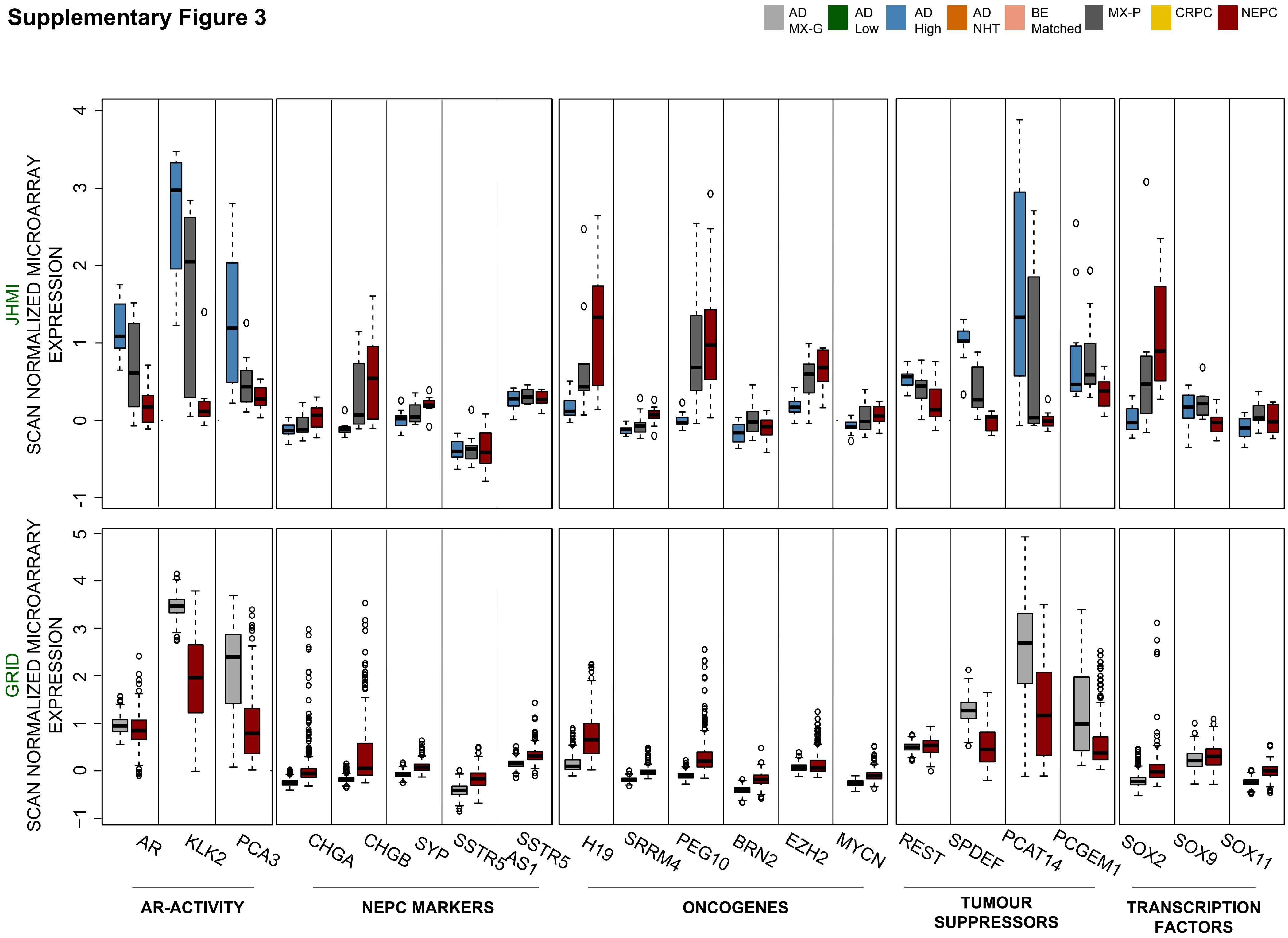

Supplementary Figure 1-3. Study cohorts and gene panel expression. Genes expression related to AR-activity (AR, KLK2, and PCA3), NEPC markers (CHGA, CHGB, SYP, SSTR5, and SSTR5-AS1), NEPC oncogenes (H19, SRRM4, PEG10, BRN2, EZH2, MYCN), NEPC tumor suppressors (REST, SPDEF, PCAT14, PCGEM1), and NEPC transcription factors (SOX family members SOX2, SOX9, and SOX11) are shown in the following cohorts VPC (n=75), WCM1 (n=37), and WCM2 (n=49) (Supplementary Figure1), WCDT (n=45) and BCCA (n=15) (Supplementary Figure 2), and microarrayed cohorts JHMI (n=33) and GRID (n=526) (Supplementary Figure 3). All sequenced cohorts had genes displayed in normalized Fragments Per Kilobase of transcript per Million mapped reads (FPKM), and all microarray cohorts' expression displayed in SCAN normalized expression. Box and Whisker plots display lower quartile, upper quartile, and median bounds of cohort expression at the box's minima, maxima, and center lines, respectively. Whisker lines display lower (bottom) and upper (top) extreme value ranges. Single data points represent outliers in a cohort. P values were calculated by unpaired two-sided Student's t-test.

Supplementary Figure 4

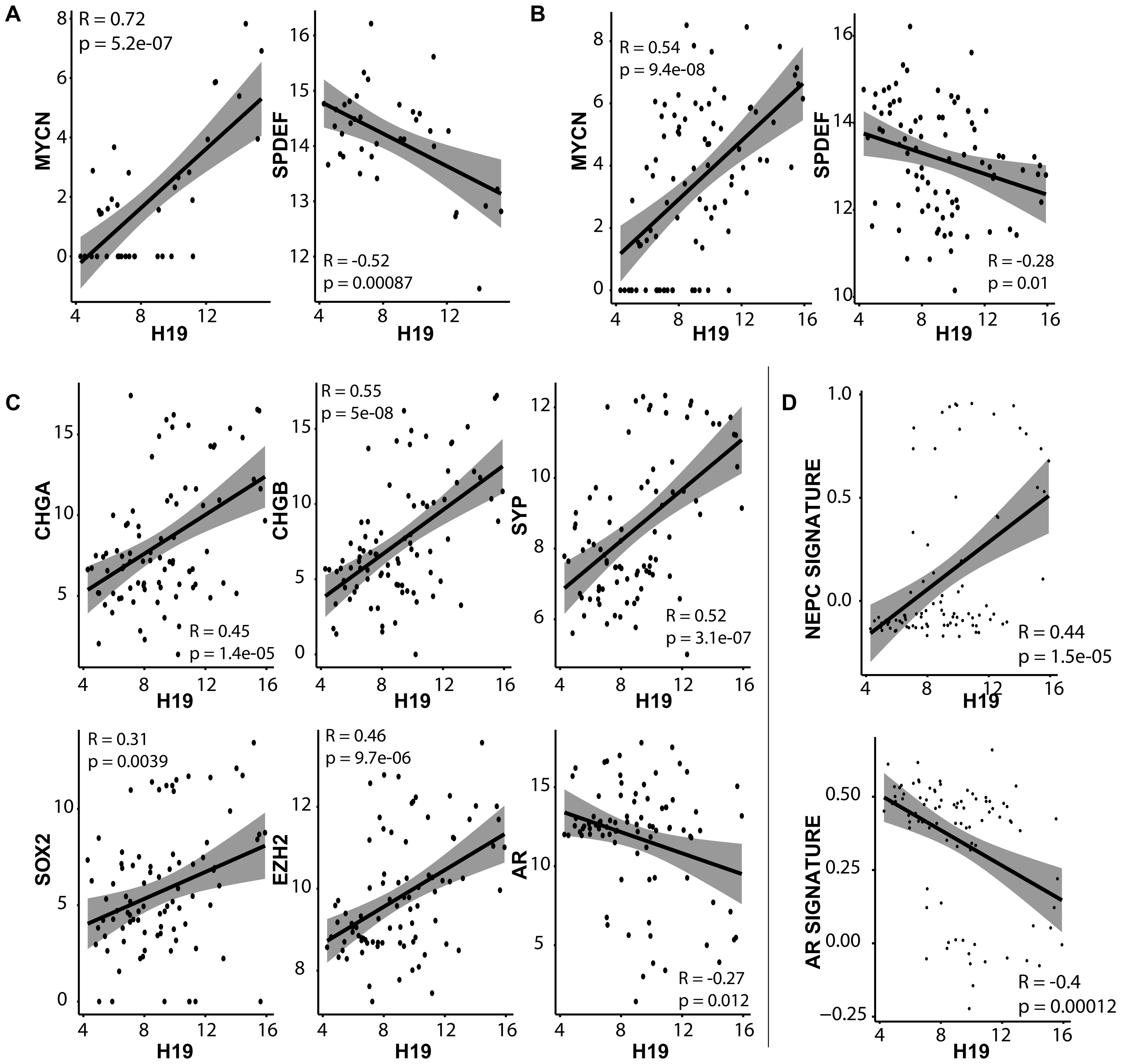

Supplementary Figure 4. H19 correlation in WCM1 and WCM2. A. H19 displays a significant positive and negative correlation to MYCN and SPDEF in WCM1, respectively. WCM1 and WCM2 are amalgamated to include CRPC phenotypes, and correlation analysis/genes from Figure 1C-D are calculated and shown in **B-D**. Similar significant correlations were observed in genes MYCN, SPDEF, CHGA, CHGB, SYP, SOX2, EZH2, AR, the NEPC gene signature, and the AR gene signature. Correlation coefficients (R) and P values (p) were calculated using a Pearson correlation statistical test. The shaded area represents confidence intervals at 95%.

Supplementary Figure 5

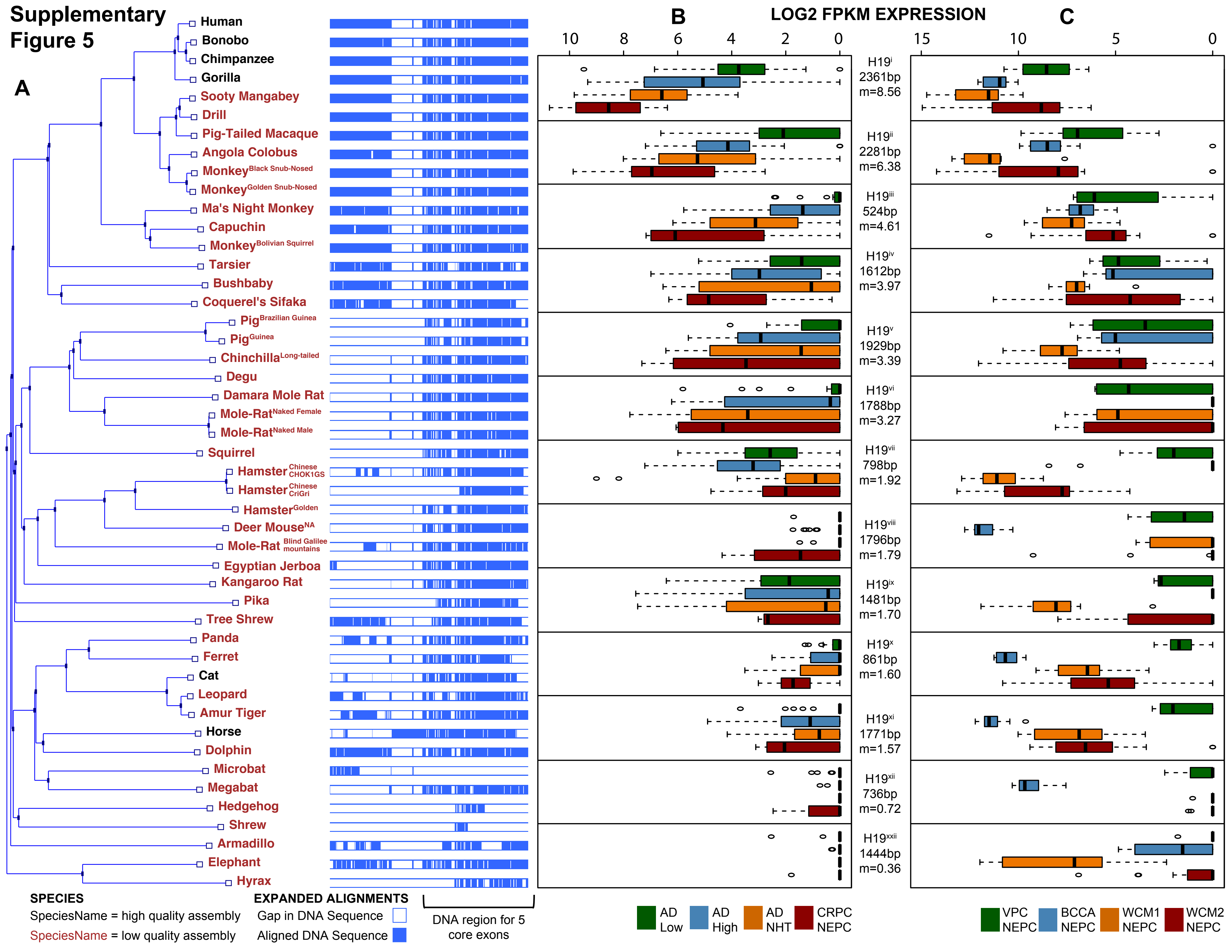

Supplementary Figure 5. H19 isoform identification and conservation. Due to the limited space and complexity of Figure 2, the complete figure is shown here. **A**. Full phylogenetic tree of Eutherian species with complete H19 DNA sequences (n=47 species of 70 analyzed). **B.** All H19 isoforms, expressed in FPKM, ranked, and displayed in decreasing order by VPC (n=75) subtype means (m). **C.** All H19 isoforms expressed in FPKM, ranked, and displayed in order by (B) for NEPC samples only in cohorts VPC, BCCA, WCM1, and WCM2 (n=40). Box and Whisker plots display lower quartile, upper quartile, and median bounds of cohort expression at the box's minima, maxima, and center lines, respectively. Whisker lines display lower (bottom) and upper (top) extreme value ranges. Single data points represent outliers in a cohort.

Supplementary Figure 6

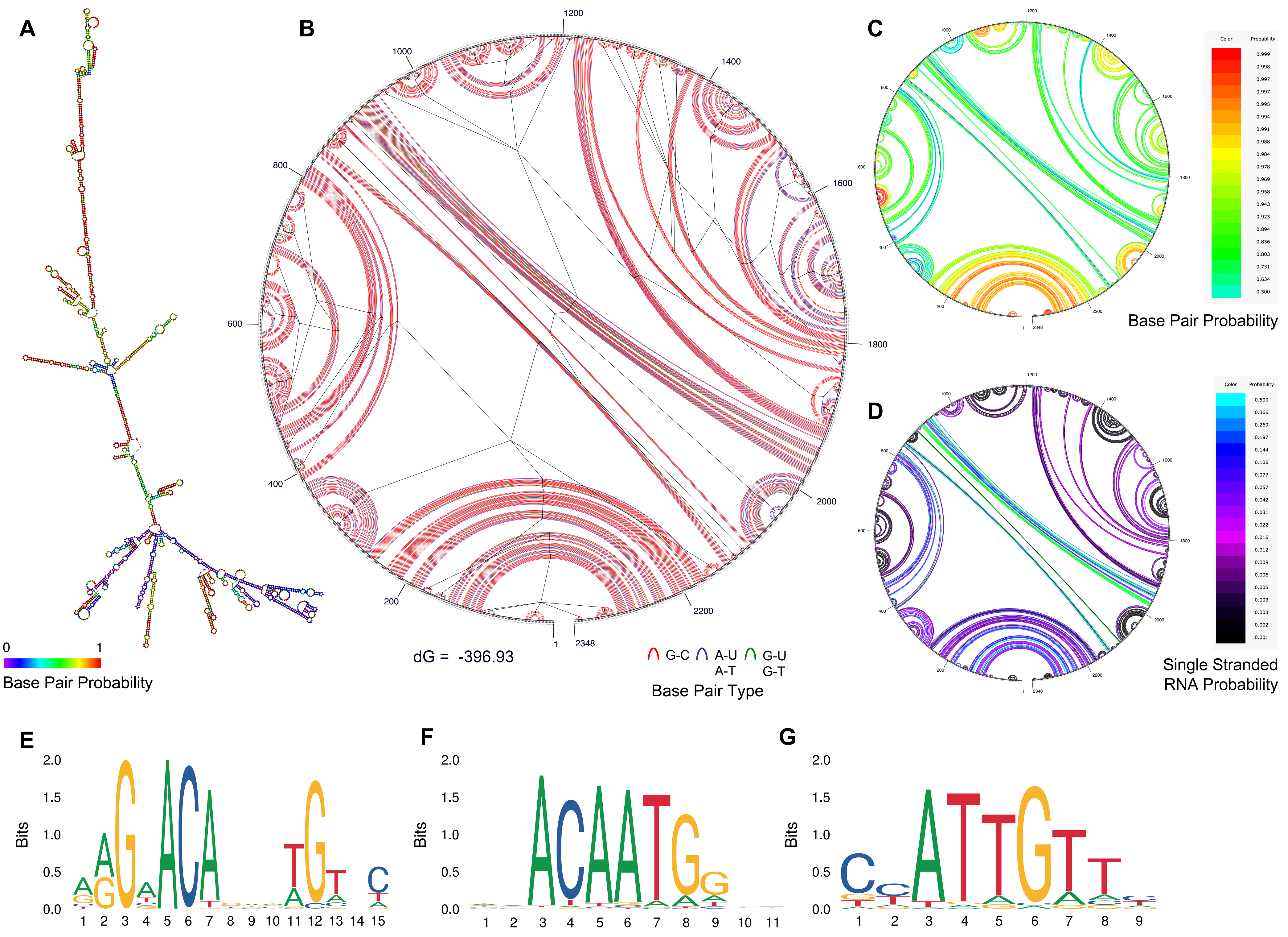

Supplementary Figure 6. H19 MFE RNA secondary structure. A. RNA fold minimum free energy (MFE) structures of H19 with each base colored by the probability of being paired with an adjacent base. **B.** Circle plot structures by the mFOLD algorithm for H19 with arcs colored based on the type of base pairing. G-C are drawn in red, A-U/A-T drawn in blue, and G-U/G-T drawn in green. Black lines represent folding angles for substructures. **C.** Circle plots with arcs colored by the probability of base pairing. **D.** Circle plots with arcs colored by the probability of single-stranded RNA. Green is approximately 0.958 probability, which is most of the arcs in (C), and purple is 0.016 probability, which is most arcs in (D). See methods for weblink with the full range of color schemes. **E-F**. Transcription factor binding motifs generated from TomTom for genes studied to regulated H19, including AR (E) and SOX2 (F).

Supplementary Figure 7

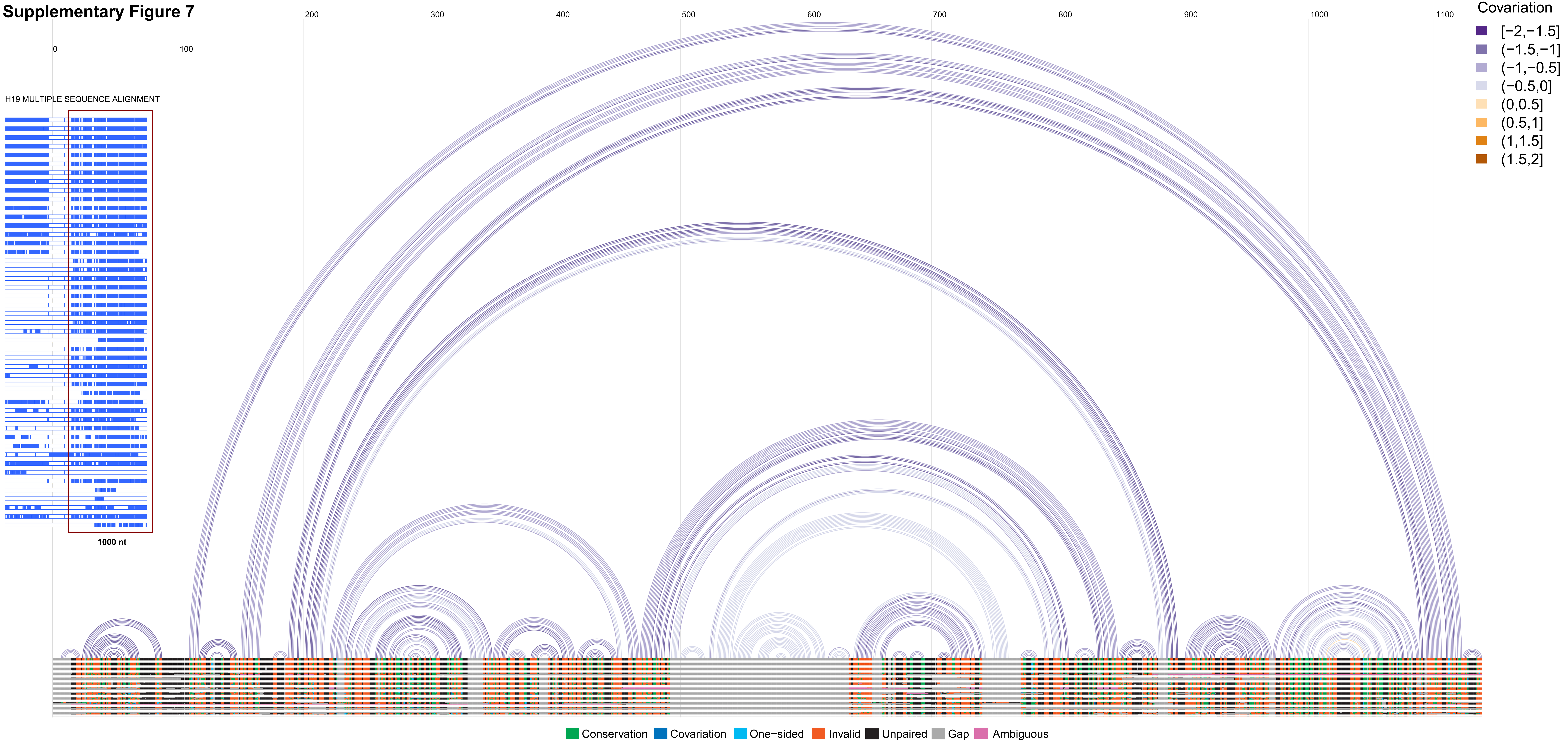

Supplementary Figure 7. H19 sequence and structure conservation across eutherian species for core exons. The highlighted box (red) of the MSA (upper left corner) spans a ~1000nt region where we narrow/zoom into for a detailed MSA (horizontal bars at the bottom) and integrate secondary structure (arcs). This region represents the core exon DNA sequences for H19. Each bar/row represents a species in the order shown from the phylogenetic tree in Figure 2A. Each arc represents a nucleotide base pairing and binding event. This region is primarily conserved despite areas with sequence differences (blue, light blue, orange, or grey). These changes do not affect H19's secondary structure, which is stable and contains little covariation across all test species. This suggests a shared function of H19 across these species.

Supplementary Figure 8

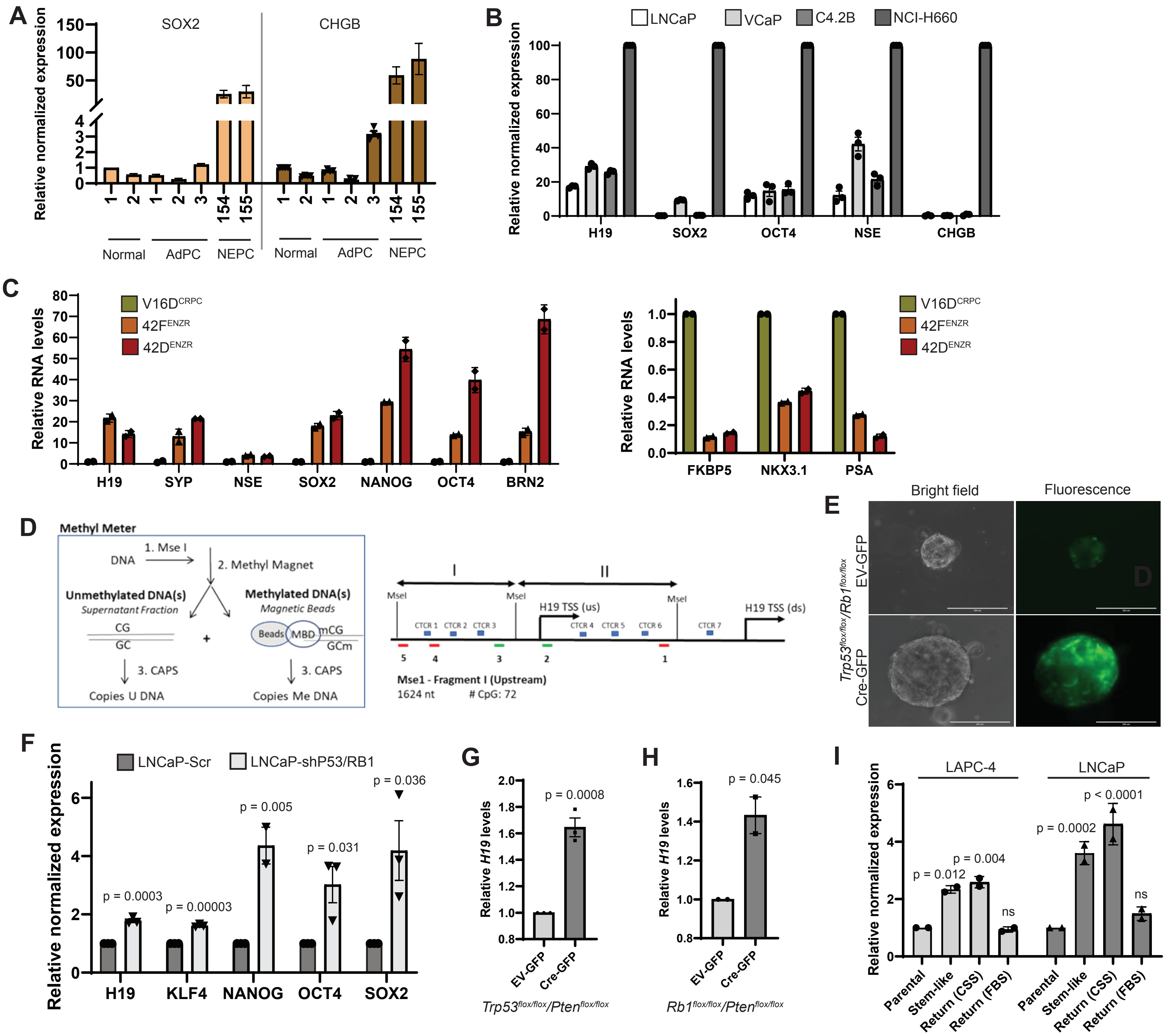

Supplementary Figure 8. H19 expression is elevated in various experimental models of NEPC. Relative RNA expression of indicated genes in **A.** organoid models derived from biopsies of NEPC (WCM154, WCM155), adenocarcinoma (AdPC 1,2), benign/normal tissue (Normal 1,2), and **B.** cell lines – LNCap, VCaP, C4-2B, NCI-H660. **C.** Relative RNA expression of the indicated NE upregulated genes (left) and AR signaling genes (right) in V16D^{CRPC}, 42D^{ENZR}, and 42F^{ENZR} cells. **D.** Schematic describing the Methylmeter assay for bisulfite free DNA methylation detection (See Supplementary Methods) (left). The *H19/IGF2* Imprinting Control Region (ICR) is indicated by the thick black line (right). This segment of H19 contains the transcription start site and seven zinc finger protein binding sites (CTCR1-7). The red and green segments indicate potential amplification targets for Coupled Abscription PCR signaling (CAPS) detection of MseI fragments I and II. The current assay measures the methylation level of the CpG island in the ICR just upstream of the H19 promoter by evaluating the 1624 bp MseI Fragment I, which contains the CTCR regions 1-3 normally methylated on the paternal strand secondary to imprinting. **E.** Representative bright field and fluorescence images of control lentivector (Lv-GFP) and Cre recombinase (Lv-Cre) transduced *Trp53*$^{fl/fl}$/*Rb1*$^{fl/fl}$ mouse (scalebar: 200 μm) (see Figure 3D). **F.** LNCaP cells were subjected to shRNA knockdown of *Trp53* and *Rb1* versus control transduction with scrambled control (Scr), and the expression of H19 and stem cell genes, Sox2, Oct4, Nanog, and Klf4 was measured. **G.** *Trp53*$^{fl/fl}$/*Pten*$^{fl/fl}$ mouse prostate organoids were transduced with Cre recombinase or control vector, and the expression of H19 was measured. **H.** *Rb1*$^{fl/fl}$/*Pten*$^{fl/fl}$ mouse prostate organoids were transduced with Cre vs. control lentivirus, and the expression of H19 was measured **I.** Relative H19 expression in LAPC-4 and LNCaP cells after incubation in charcoal-stripped serum (CSS) to induce stem-like NEPC followed by incubation in FBS to return to the adenocarcinoma phenotype. Changes were as compared to parental cells. Data are mean +/- SEM (**F, G**) or mean +/- SD (**A-C, H, I**) of biologically independent triplicate determinations. P values were calculated by unpaired two-tailed student t-test.

Supplementary Figure 9

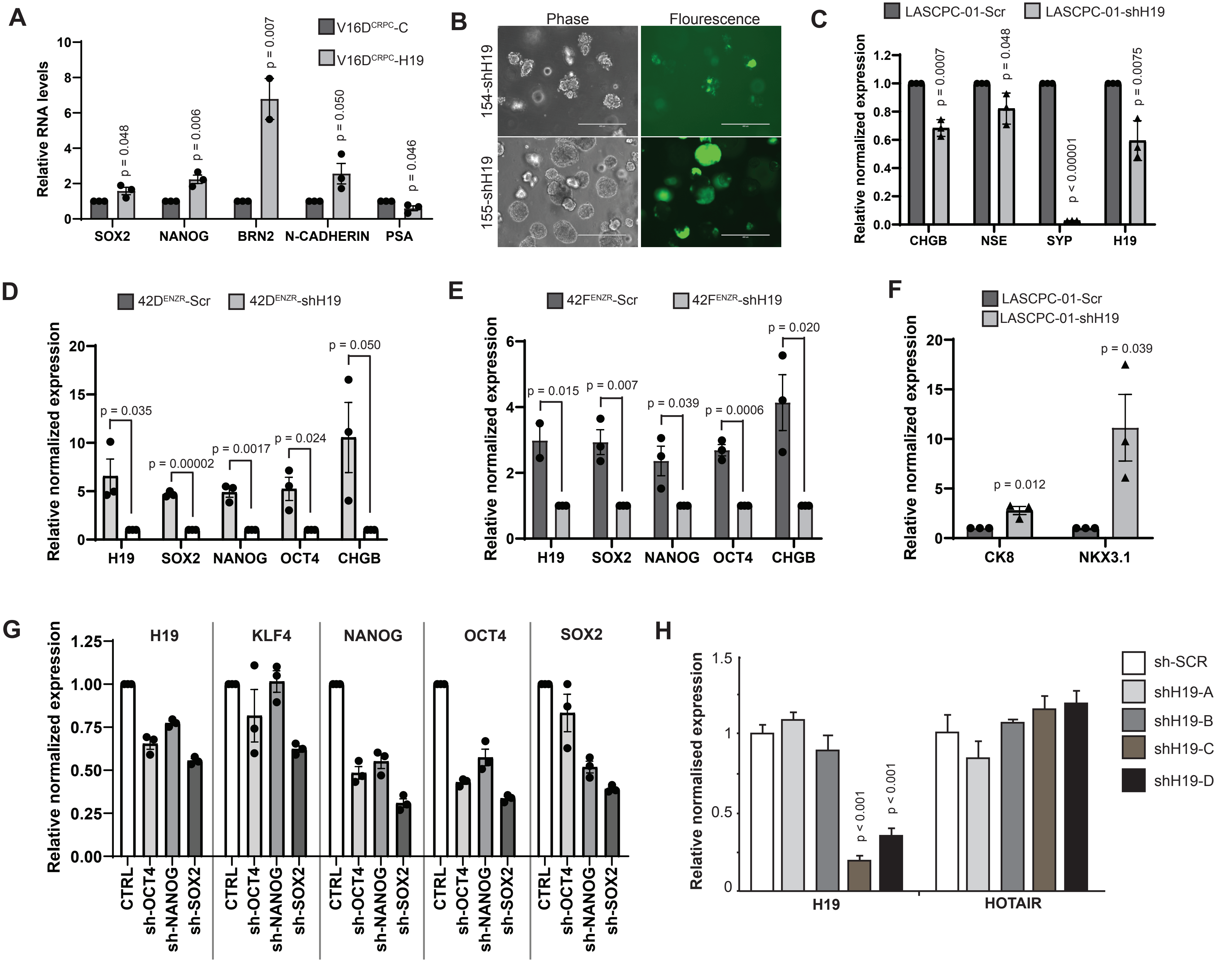

Supplementary Figure 9 Continued

I

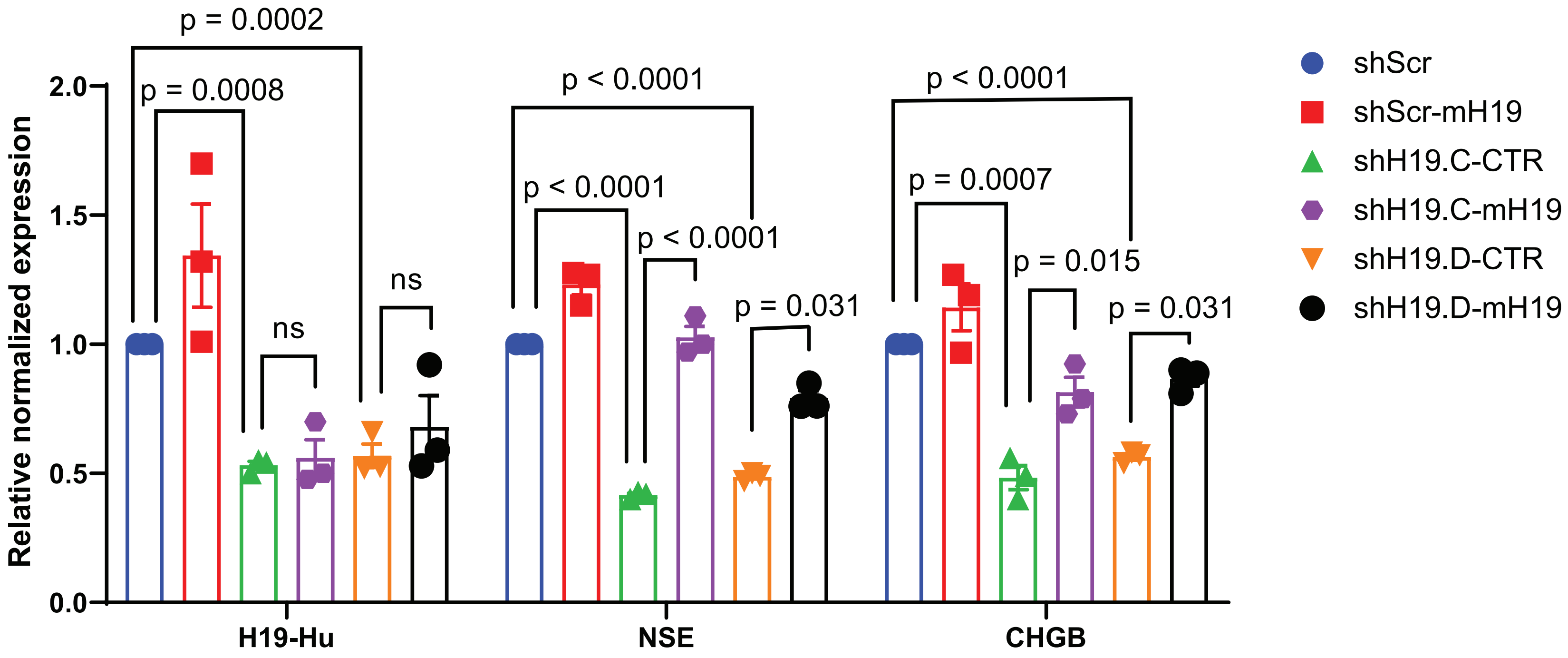

J

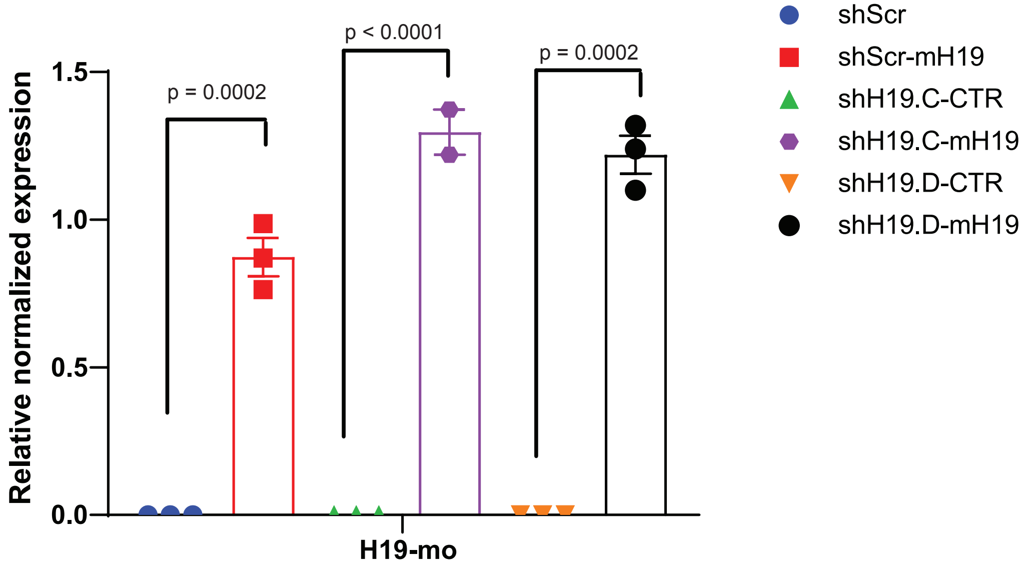

Supplementary Figure 9. H19 regulates the expression of stem cell genes and NE markers. A. Relative RNA expression of the indicated genes in V16D^{CRPC} cells overexpressing H19 (V16D-H19) versus control (V16D-C). **B.** Representative images of NEPC organoids OWCM154-shH19 and OWCM155-shH19 (described in Figure 4C) transduced with shRNA to H19 (scalebar: 400 µm). **C-E.** Expression of the indicated genes in LASCPC-01 (C), 42D^{ENZR} (D), and 42F^{ENZR} (E) after H19 knockdown compared to control Scr transfection. **F.** Relative RNA expression of luminal markers (CK8, NKX3.1) after H19 knockdown in LASCPC-01 cells (LASCPC-01-shH19) compared to control LASCPC-01-Scr cells. **G.** LNCaP cells were transfected with either shRNA to OCT-4, NANOG, and SOX2 or scrambled control (Scr), and the mRNA expression of H19, KLF-4, OCT-4, SOX2, and Nanog was measured by qRT-PCR. The result represents one independent experiment performed in triplicate. **H.** 42D^{ENZR} cells were transfected with either shRNA to H19 (shH19 A, B, C, and D) or Scr, and then mRNA expression of H19 and HOTAIR was measured by qRT-PCR to ascertain the specificity of H19 knockdown. **I-J.** 42D^{ENZR} cells stably expressing shRNA to H19 or Scr were re-transduced with either Ctr or murine H19, and the mRNA expression of H19, NSE, and CHGB was measured by qRT-PCR. Data are mean +/- SEM of biologically independent triplicate determinations. P values were calculated by unpaired two-tailed Student's t-test (**A, C-H, I**) or Tukey's multiple comparisons test (J).

Supplementary Figure 10

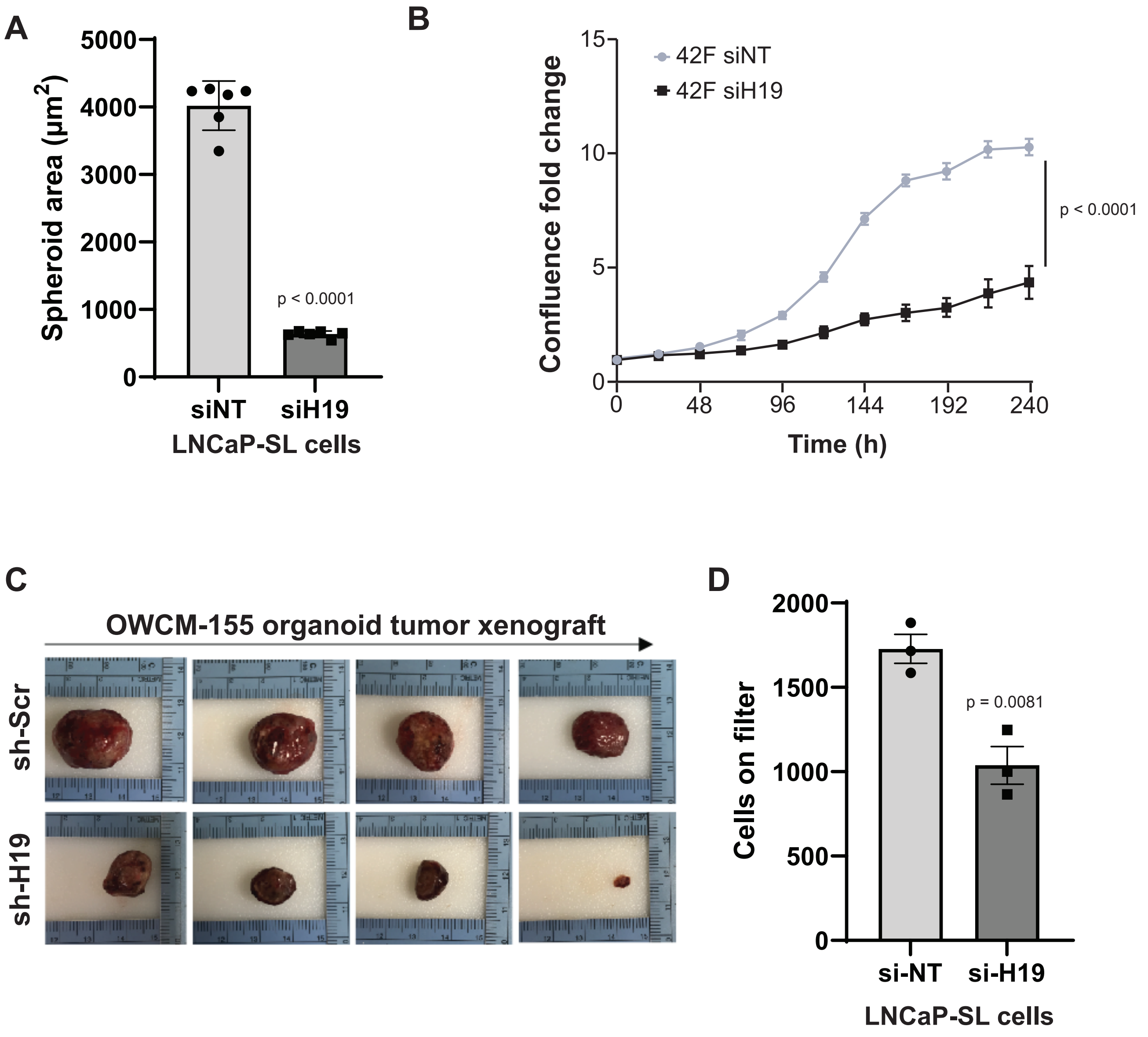

Supplementary Figure 10. Elevated H19 is essential for proliferation and invasion of NEPC. A. LNCaP-SL spheroid area after transient knockdown of H19(si-H19) compared to control transfected cells (si-NT). **B.** Proliferation of $42F^{ENZR}$ cells transduced with siH19 versus siNT control. **C.** Photographic images of tumor xenografts of OWCM-155 organoids transduced with shScr control or shH19 11-week post-injection into NSG mice. **D.** Transwell migration of NE-like LNCaP-SL cells with H19 knockdown (si-H19) as compared to control transfected cells (si-NT). Data are mean +/- SEM of n=6 (**A**) and n=3 (**B, D**) biologically independent determinations. P values were calculated by unpaired two-tailed student t-test (**A, D**) or two-way ANOVA (**B**).

Supplementary Figure 11

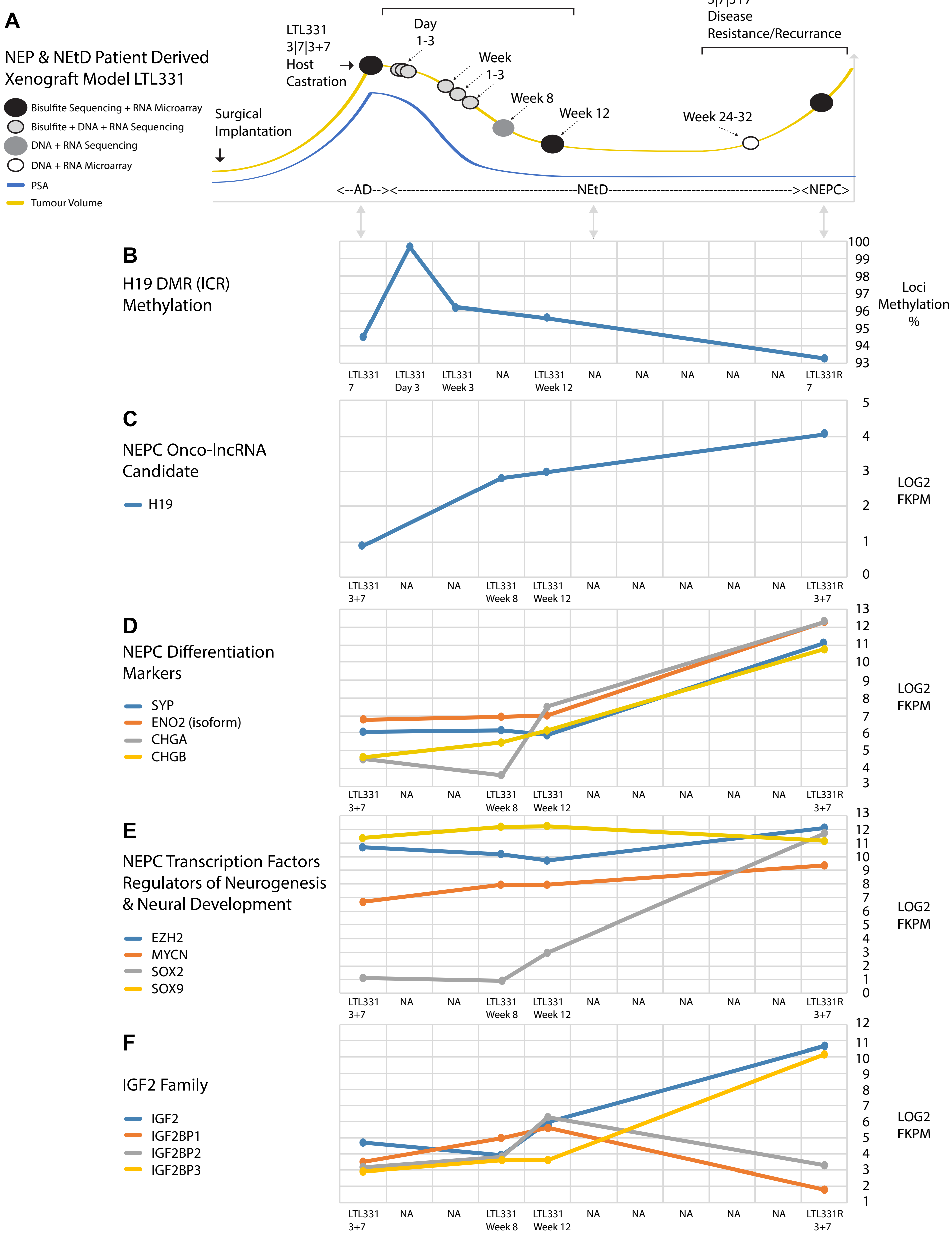

Supplementary Figure 11. Patient-Derived Xenograft (LTL331) Time Series of NEtD with Transcriptomic Context. A. A time series model of NEtD in LTL331/LTL331R with various next-generation sequence profiling (RNA, DNA, and Methylation). **B.** Methylation status for the ICR locus. **C-F.** Expression levels showing changes in H19 (C), NEPC differentiation markers (SYP, ENO2/NSE, CHGA, and CHGB) **(**D**)**, NEPC transcription factors (SOX2, SOX9, EZH2, NMYC) **(**E**)**, and IGF2 family members (F). Results (**B-C**) are derived from n=1 sequenced xenograft model/sample.

Supplementary Figure 12

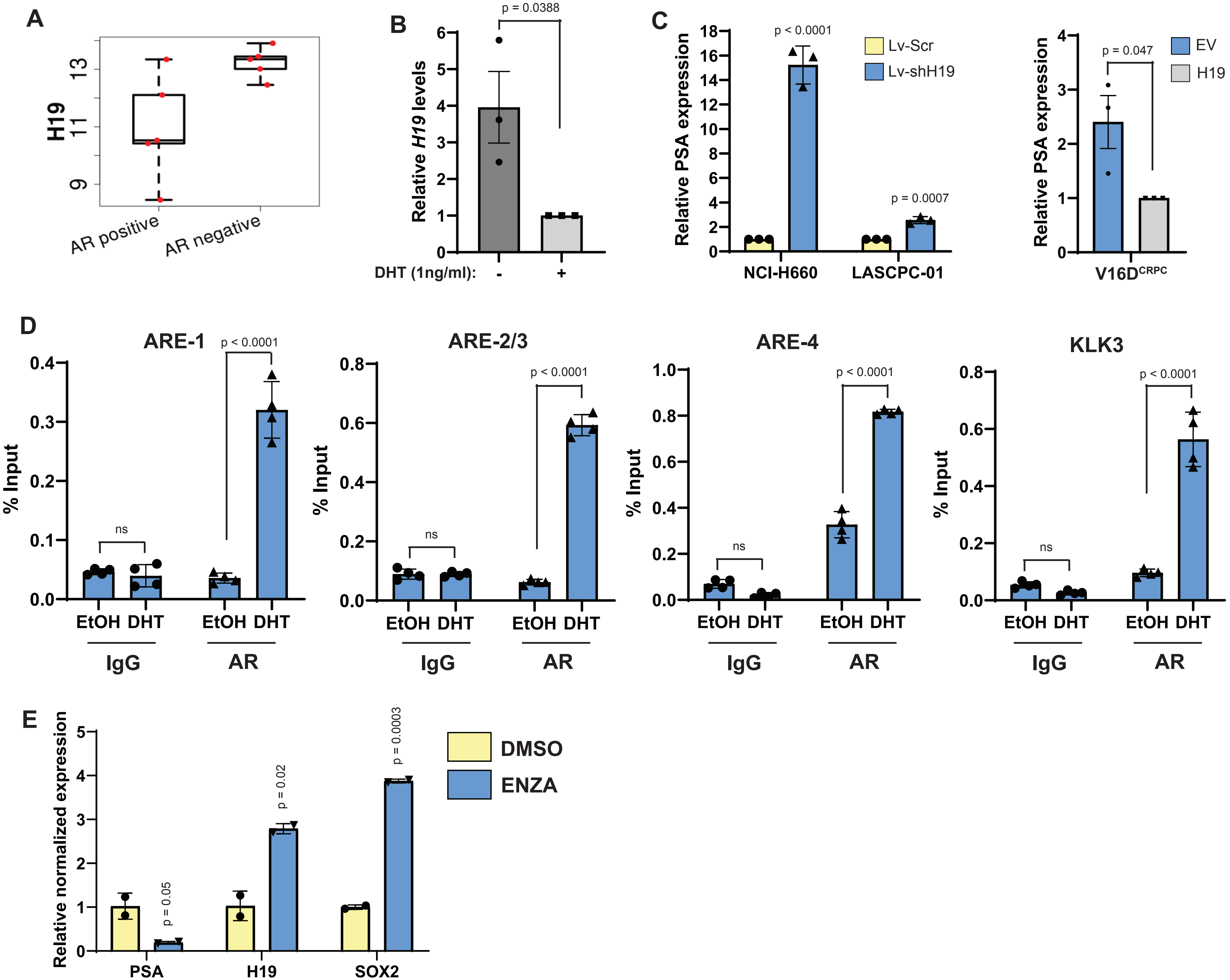

Supplementary Figure 12. Androgen regulation of H19 expression. A. H19 expression in the LuCaP series of human prostrate PDX tumors (GSE93809) separated by androgen receptor (AR) status. Box and Whisker plots display lower quartile, upper quartile, and median bounds of cohort expression at the box's minima, maxima, and center lines, respectively. Whisker lines display lower (bottom) and upper (top) extreme value ranges. Single data points represent outliers in a cohort. **B.** H19 expression in murine prostate organoids upon DHT removal (DHT-) compared to cells cultured in DHT-containing media (DHT+, 1 ng/ml). **C.** PSA mRNA expression in NCI-H660 and LASCPC-01 after transduction with lentiviral (Lv) shH19 when compared to control Lv-Scr. PSA expression in V16D^{CRPC} and Hu-AdPC organoids with H19 or empty vector (EV) overexpression. **D.** ChIP-qPCR of C4-2B cells showing enrichment of AR bound to the H19 promoter after DHT (10 nM) versus ethanol (EtOH) control treatment. The KLK3 promoter was used as a positive control. **E.** C4-2B cells were treated with ENZA (10 μM) for five days, and the expression of PSA, H19, and SOX2 was measured. Data are mean +/- SEM of n=3 (**B, C, E**), and n=4 (**D**) biologically independent determinations. P values were calculated by unpaired two-tailed Student's t-test (**B, C, E**) or Sidak's multiple comparisons test (**D**).

Supplementary Figure 13

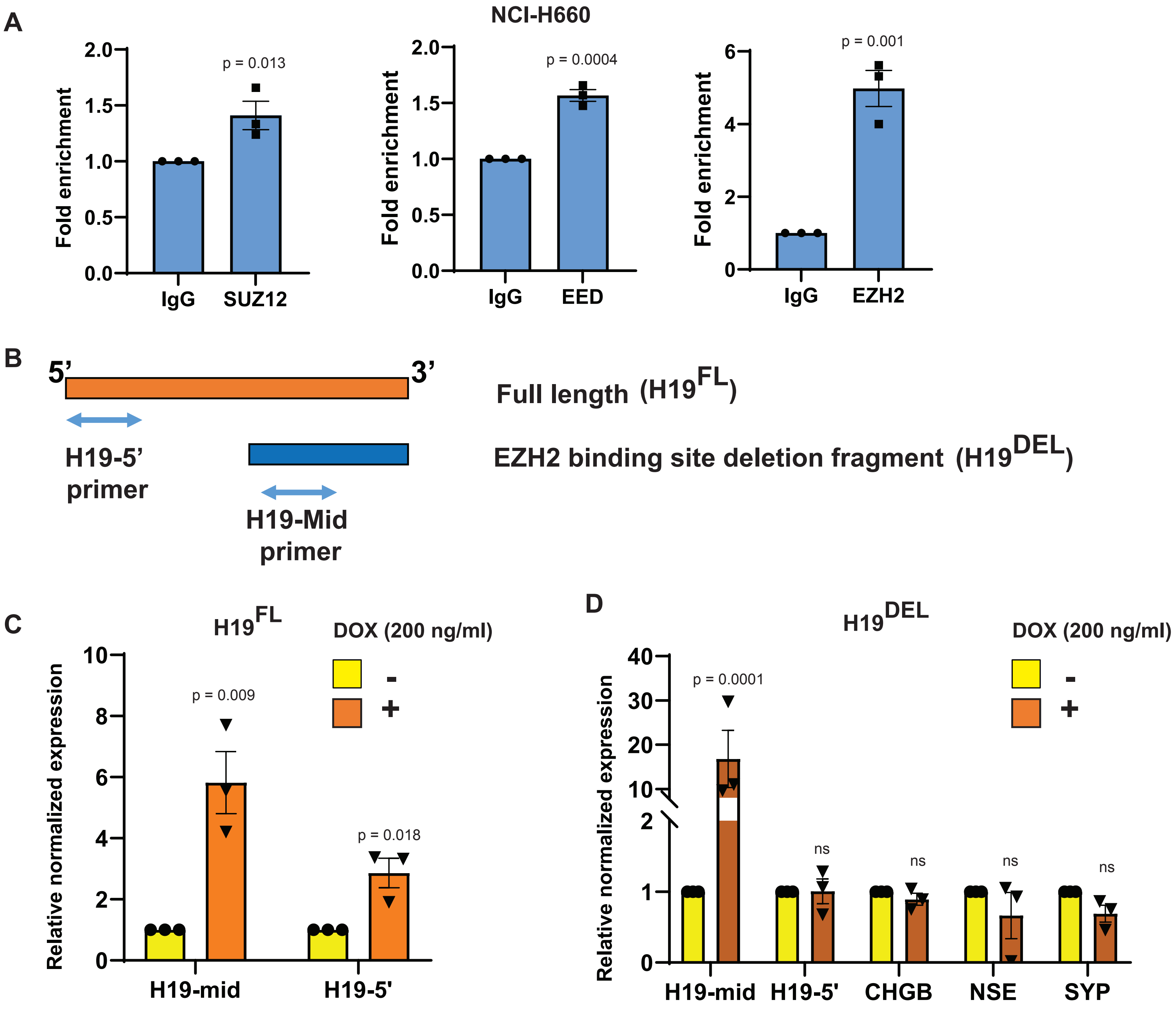

Supplementary Figure 13. The full length of H19 is critical to driving NE genes. A. Fold enrichment after RNA immunoprecipitation of PRC2 complex members EZH2 (bottom), EED (middle), SUZ12 (top) with H19 in NCI-H660 cells. Data are mean+/- SD. **B.** The schema shows full-length H19 and an EZH2-binding site deletion fragment cloned into a doxycycline (DOX) inducible Tripz vector. The full-length fragment is 2.3kb, while the shorter fragment is 1260 bp to 3' end. The double-head arrows indicate the specific primer pair regions used to detect one or both H19 constructs. **C.** The expression vectors in (A) were transduced into LNCaP cells, which were treated with DOX (200ng/ml) or DMSO for 48h and the expression measured by the two sets of primers identified above. **D.** The expression of H19 and NE genes (NSE, CHGB, and SYP) in LNCaP cells expressing $H19^{DEL}$ treated with DOX (200ng/ml) or DMSO for 48h. The $H19^{DEL}$ expression was detected with the H19-5' and H19-Mid primers. Data are mean +/- SEM of n=3 biologically independent determinations. P values were calculated by unpaired two-tailed Student's t-test (**A, C**) or Sidak's multiple comparisons test (**D**).

Supplementary Figure 14

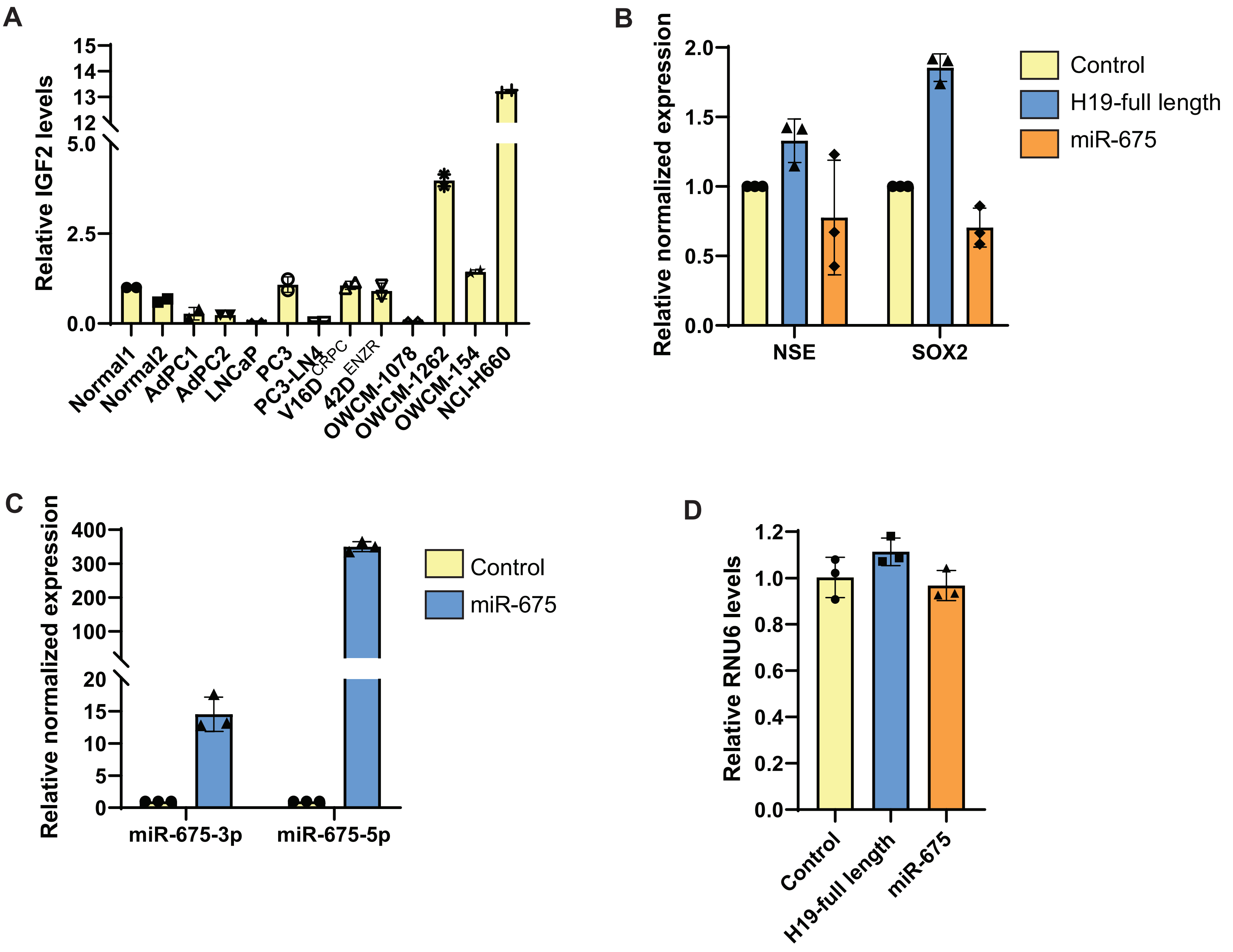

Supplementary Figure 14. IGF2 expression and role of miR-675 in H19 mediated NEtD. A. Relative RNA expression of IGF2 in organoid models derived from biopsies of NEPC (OWCM154, OWCM1262, and OWCM1078), adenocarcinoma (AdPC 1,2), benign/normal tissue (Normal 1,2), and in various prostate cancer cell lines (PC3, PC3-LN4, V16D^{CRPC}, 42D^{ENZR}, LNCAP, and NCI-H660). **B-D.** Murine prostate organoids were transduced with Control, H19 full length, and miR-675 encoding H19 fragment (see Supplementary Methods), and the expression of NSE, SOX2, miR-675, and RNU6 was measured by qRT-PCR. Data are mean +/- SD of n=3 replicates. The result represents biological replicate (**A**) and one independent experiment performed in triplicate (**B-D**).

Supplementary Figure 15

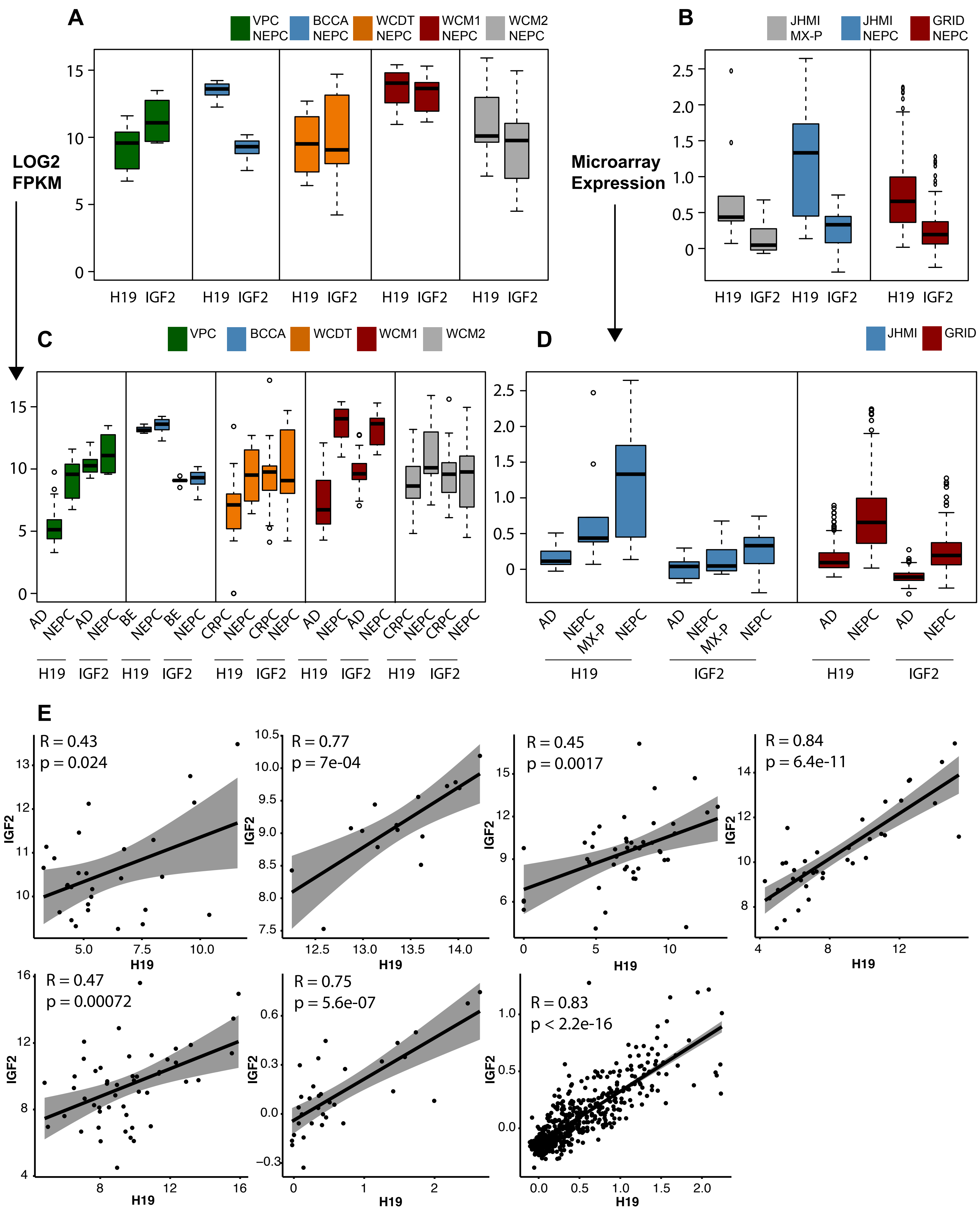

Supplementary Figure 15. Clinical sample expression and correlation for H19 and IGF2. A-B. Pairwise expression of H19-IGF2 in VPC (n=75), BCCA (n=15), WCDT (n=45), WCM1 (n=37), WCM2 (n=49), JHMI (n=33), and GRID (n=526) cohorts showing relatively higher expression of IGF2 compared to H19 in NEPC samples only. **C-D.** Pairwise expression of H19-IGF2 in VPC (n=75), BCCA (n=15), WCDT (n=45), WCM1 (n=37), WCM2 (n=49), JHMI (n=33), and GRID (n=526) cohorts showing relatively higher expression of H19 over IGF2 in NEPC samples compared to AD/CRPC. Box and Whisker plots display lower quartile, upper quartile, and median bounds of cohort expression at the box's minima, maxima, and center lines, respectively. Whisker lines display lower (bottom) and upper (top) extreme value ranges. Single data points represent outliers in a cohort. **E.** H19 expression shows a significant positive correlation to IGF2 expression in NEPC samples of (left to right) VPC (n=75), BCCA (n=15), WCDT (n=45), WCM1 (n=37), WCM2 (n=49), JHMI (n=33), and GRID (n=526) cohorts. Correlation coefficients (R) and P values (p) were calculated using a Pearson correlation statistical test. The shaded area represents confidence intervals at 95%.
